# Biomechanical effect of endplate defects on the intermediate vertebral bone in consecutive two-level anterior cervical discectomy and fusion: a finite element analysis

**DOI:** 10.1186/s12891-023-06453-3

**Published:** 2023-05-22

**Authors:** Jiarui Zhang, Wenzhao Chen, Rui Weng, De Liang, Xiaobing Jiang, Hongheng Lin

**Affiliations:** 1 Guangzhou University of Chinese Medicine, Guangzhou, 510405 China; 2 Department of Spine Surgery, The Third Affiliated Hospital of Guangzhou University of Chinese Medicine, Guangzhou, 510378 China; 3 Department of Spine Surgery, The First Affiliated Hospital of Guangzhou University of Chinese Medicine, Guangzhou, China

**Keywords:** Cervical spine, Endplate defect, ACDF, Vertebral collapse, Finite element analysis

## Abstract

**Background:**

Intermediate vertebral collapse is a newly discovered complication of consecutive two-level anterior cervical discectomy and fusion (ACDF). There have been no analytical studies related to the effects of endplate defects on the biomechanics of the intermediate vertebral bone after ACDF. This study aimed to compare the effects of endplate defects on the intermediate vertebral bone biomechanics in the zero-profile (ZP) and cage-and-plate (CP) methods of consecutive 2-level ACDF and to determine whether collapse of the intermediate vertebra is more likely to occur using ZP.

**Methods:**

A three-dimensional finite element (FE) model of the intact cervical spine (C2–T1) was constructed and validated. The intact FE model was then modified to build ACDF models and imitate the situation of endplate injury, establishing two groups of models (ZP, IM-ZP and CP, IM-ZP). We simulated cervical motion, such as flexion, extension, lateral bending and axial rotation, and compared the range of motion (ROM), upper and lower endplate stress, fusion fixation device stress, C5 vertebral body stress, intervertebral disc internal pressure (intradiscal pressure, or IDP) and the ROM of adjacent segments in the models.

**Results:**

There was no significant difference between the IM-CP model and the CP model in the ROM of the surgical segment, upper and lower endplate stress, fusion fixation device stress, C5 vertebral body stress, IDP, or ROM of the adjacent segments. Compared with the CP model, the endplate stress of the ZP model is significantly higher in the flexion, extension, lateral bending and axial rotation conditions. Compared with the ZP model, endplate stress, screw stress, C5 vertebral stress and IDP in IM-ZP were significantly increased under flexion, extension, lateral bending and axial rotation conditions.

**Conclusions:**

Compared to consecutive 2-level ACDF using CP, collapse of the intermediate vertebra is more likely to occur using ZP due to its mechanical characteristics. Intraoperative endplate defects of the anterior lower margin of the middle vertebra are a risk factor leading to collapse of the middle vertebra after consecutive 2-level ACDF using ZP.

**Supplementary Information:**

The online version contains supplementary material available at 10.1186/s12891-023-06453-3.

## Introduction

Anterior cervical discectomy and fusion (ACDF) is a classic operation for the treatment of cervical spondylosis. Since it was first reported by Robinson and Smith in 1958, ACDF has been widely used in the clinic because of its minimal invasiveness, high fusion rate and effective spinal cord decompression [1–3]. At present, the most commonly used fixation methods for ACDF are divided into two categories: the cage-and-plate construct (CP) and zero-profile anterior interbody fusion (ZP). Both the CP and ZP methods achieve the effect of fusion between the upper and lower vertebral bodies by removing the surgical segmental intervertebral discs and placing the fusion device [4]. For two-segment ACDF, it has not been clear whether to use the ZP or CP method. Both fixation methods are widely used in the clinic. Previous studies [5] have found that the middle vertebral body of the fixed segment is prone to collapse in patients treated with ZP fixation but not in patients treated with CP fixation. Some of these studies noted that the change in blood supply in the middle vertebral body of the fixed segment, excessive stress conduction through the endplate and damage to the anterior margin of the endplate on the fusion segments may be risk factors leading to the collapse of the middle vertebral body.

The endplate is a kind of cortical outer structure located at the upper and lower parts of the vertebral body. Because of the small elastic modulus of cancellous bone, the endplate plays a major role in the compressive strength of the vertebral body. In intervertebral fusion surgery, retention of the endplate can effectively reduce the settlement of implants and maintain the balance of the intervertebral mechanical relationship. To date, Modic changes and structural abnormalities of the lumbar endplate have been demonstrated to be related to lumbar instability and degeneration [6]. However, there have been relatively few studies on the relevance between Modic changes and structural abnormalities of the cervical endplate and clinical diseases. In recent years, studies by Harada et al. [7] and Baker et al. [8] revealed that cervical endplate abnormalities can affect the preoperative and postoperative cervical sequence parameters, aggravate postoperative pain following, and increase ASD and reoperation rates. Although the impact of endplate abnormalities on the mechanical stability of the cervical spine was not explored, these studies indicated the importance of the morphological stability of the cervical endplate. At the same time, the literature [9] also supports the viewpoint that the cervical endplate plays a substantial role in maintaining the mechanical stability of the cervical spine. However, there have been few reports on the effect of intraoperative damage to the anterior margin of the endplate on the effectiveness and long-term results of surgery, and there have been no relevant biomechanical studies.

Therefore, in this study, three-dimensional finite element (FE) models were constructed to simulate two consecutive segments undergoing ACDF using CP and ZP. The postoperative stress distribution characteristics of the cervical spine in the two cases were analyzed and compared. According to the stress distribution characteristics, an analysis was performed to determine whether middle vertebral body collapse was more likely to occur in the two consecutive segments of the ACDF using the ZP fixation method. On this basis, the model of the intraoperative damage of the endplate at the anterior-inferior margin of the vertebral body was further simulated and compared with the model of the intact endplate to study the effect of the damage of the endplate at the anterior-inferior margin of the vertebral body on the postoperative stress changes of the two consecutive segments in the ACDF and to analyze whether the defect of the endplate bone at the anterior-inferior margin of the vertebral body is more likely to lead to intermediate vertebral collapse in patients treated with two-level ACDF.

## Materials and methods

### Models

The geometric reconstruction of the three-dimensional FE model of the cervical spine was based on a healthy volunteer, with tumors, deformities and spine-related diseases excluded. The present C2-T1 model was developed on the basis of previous C3-C7 models [10]. Geometric details of the bony structure were obtained by high-resolution computed tomography (CT, with a slice thickness of 0.625 mm). The medical engineering software Mimics 19.0 was used to extract the geometry of the model and establish the 3D model. Then, Geomagic Studio software was used to smooth the surface of the vertebral body, generate the geometric solid of the model, and output the results as an STP file. The intervertebral disc structure was obtained by magnetic resonance imaging (MRI), and the intervertebral disc tissue was reconstructed as described above. Subsequently, the STP files were imported into Solidworks2014 software for combination and assembly and finally exported to HyperMesh 2019 software.

The skeletal structure was modeled by a combination of the shell and solid elements. The cortical bone regions of the C2-C7 vertebral bodies were all modeled by a triangular shell element to accurately fit the anatomical features, and the thickness was reconstructed according to autopsy data in the literature [11]. The whole cortical bone of each vertebral body was filled, and the cancellous bone in the cortical bone was modeled by a tetrahedral element, with the cortical bone and the cancellous bone as co-nodes. The articular cartilage model and the 0.5-mm-thick endplate model were generated according to the vertebral bone markers and the anatomical structure [12]. The intervertebral disc consisted of the annulus fibrosus matrix, nucleus pulposus and annulus fibrosus fibers, which were modeled by the hexahedral element and the quadrilateral membrane element, respectively [13]. The intervertebral disc was bound to the adjacent vertebral endplate. The modeling details of the fiber layer were selected with reference to the method introduced by Östh and Shen [14]. The cartilage endplate was bound to the upper and lower surfaces of the intervertebral disc. The ligament structures in the model, including each anterior longitudinal ligament (ALL), posterior longitudinal ligament (PLL), ligamentum flavum (LF), interspinous ligament (ISL) and articular capsule ligament (FCL) of C2-T1, used quadrilateral membrane element attributes based on the anatomical structure. The articular cartilage was modeled by the quadrilateral shell element, and the mesh types are shown in Table 1.

**Table 1:** Summary of material and element properties used for the FE model

Component	Element type	Constitutive model	Constitutive model (MPa)	Shell thickness (mm)
Cortical bone	Triangular shell	Elastic–Plastic	$E$= 12,000 $G$= 1250 $\sigma$_yield_ = 189.8 $v$= 0.3	C1-C3 = 0.51 C4 = 0.55 C5 = 0.62 C6 = 0.66 C7 = 0.70 T1 = 0.30
Cancellous bone	Tetrahedral	Elastic–Plastic	$E$= 442 $G$= 51 $v$= 0.3	N/A
Bony endplate	Triangular shell	Elastic–Plastic	$E$= 5600 $G$= 300 $\sigma$_yield_ = 36.7 $v$ = 0.3	0.5
Nucleus pulpous	Hexahedral	Incompressible fluid	$k$= 1720	N/A
Annulus fibrosus matrix	Hexahedral	Mooney-Rivin	$A$= 0.18 (constants) $B$ = 0.045 (constants)	N/A
Annulus fibrosus fibers	Quadrilateral membrane	Orthotropic nonlinear elastic	$E$_c_ = 0.01–7.1 $v$_ab_ = 0.3	0.25
ligaments	Quadrilateral membrane	Orthotropic nonlinear elastic	$E$_c_ = 0.4–23.3 $v$_ab_ = 0.3 $v$_ca_ = $v$_cb_ = 0.49	ALL = 1.5 PLL = 1.7 LF = 1.3 FCL = 1.1 ISL C2-C5 = 1.4 ISL C6-T1 = 1.0

*E* Elastic modulus, *G* Shear modulus, *σ*_*yield*_ Yield stress, *v* Poisson’s ratio, *k* Bulk modulus, *Ec* Curve of elastic modulus, *N/A* Not applicable

**Fig. 1:**
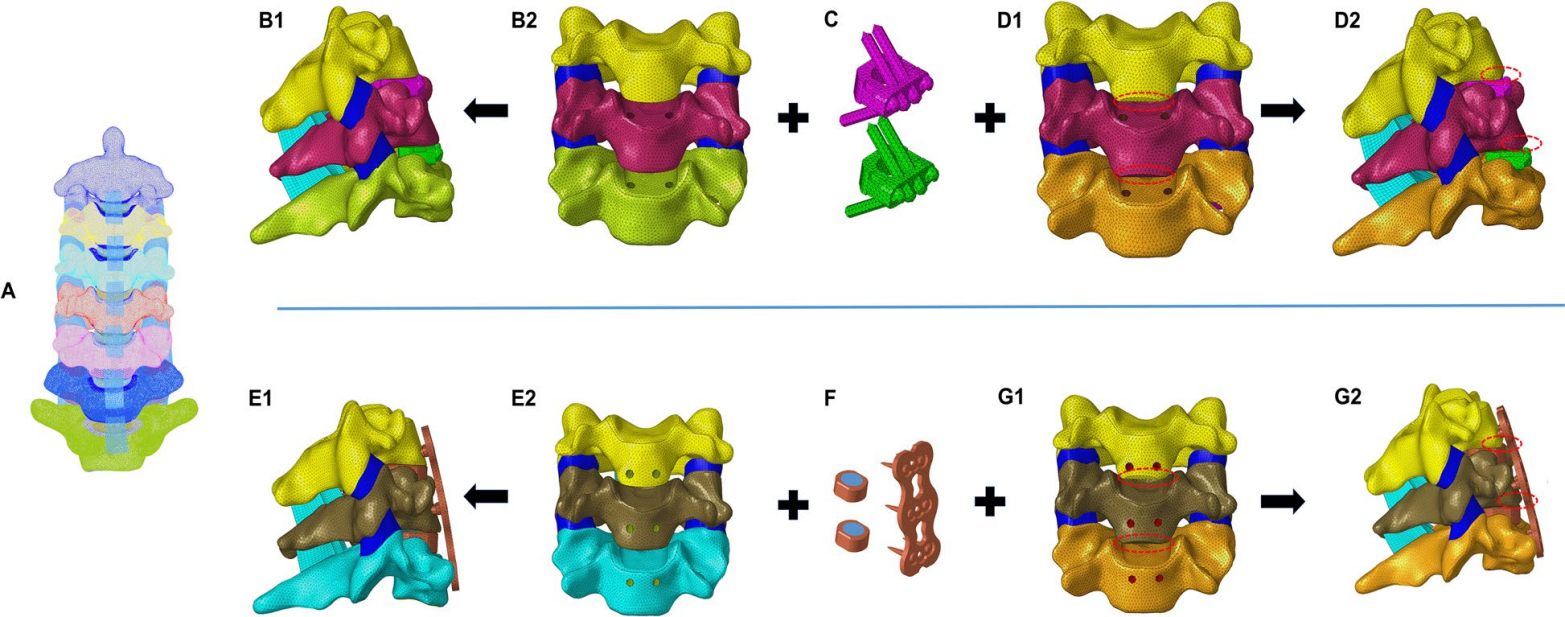
**A** A three-dimensional finite element (FE) model was developed. **B-G ** Two-group models of two-level anterior cervical discectomy with fusion (ACDF) using different internal fixation were established. **B-C** C4**-**C6 implanted by the zero-profile device (ZP). **C-D** C4**-**C6 with the endplate injury implanted by the ZP. **E-F** C4**-**C6 implanted by the cage-plate device (CP). **F-G** C4**-**C6 with the endplate injury implanted by the CP

**Fig. 2:**
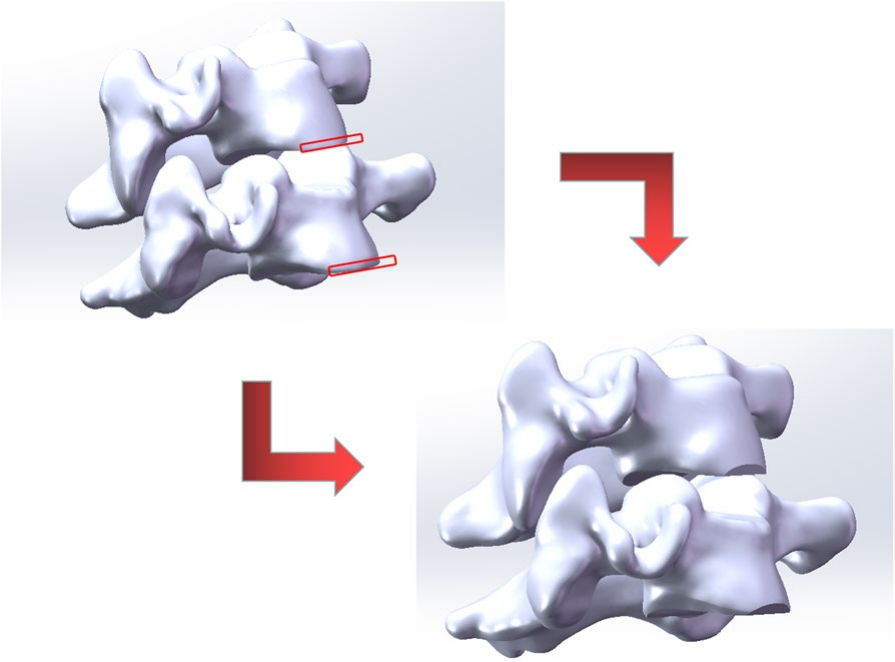
C4 and C5 vertebral body’s resection for lower endplate in the operation was simulated and established the injury model (IM) of endplate

Based on the normal cervical FE model (Fig. 1A), steel plates, screws, cages and Zero-P devices were drawn on the basis of the model, and the anterior longitudinal ligaments, posterior longitudinal ligaments, intervertebral discs and cartilage endplates of C4-C6 double segments were removed at the same time. The ZP system was fixed with 4 screws (3 mm in diameter and 16 mm in length), and the CP system was fixed with 6 screws and steel plates (the steel plates were 16 mm wide and 2 mm thick, and the fixing screws were 4 mm in diameter and 14 mm in length) (Fig. 1). See Table 2 for the material definitions of various implants. In this study, the fixed segments were C4–C6, and the simulated damaged endplates were the C4 lower endplate and the C5 lower endplate. In SOLIDWORKS software, the resection tool was used to resect approximately 2 mm from the lower endplates of the C4 and C5 vertebral bodies at an angle of 15° (Fig. 2), which simulated the intraoperative situation. Four independent two-segment (C4/5, C5/6) anterior cervical models were created by combining the internal fixation device and the cervical model (Fig. 1).

**Table 2:** Mechanical properties used to simulate fixation devices of the FE models

	$E$(MPa)	$v$
Cage(PEEK)	3600	0.3
Plate	110,000	0.3
Screw	110,000	0.3

### Material properties

The definitions of the materials of the model are all based on the previous literature, as shown in Table 1. The cortical, cancellous and bony endplates of the vertebral body are all defined as an isotropic elastoplastic material [15–17]. Among them, the nucleus pulposus model was modeled by elastic fluid property parameters, while the annulus fibrosus matrix and the annulus fibrosus fibers had hyperelastic and orthogonal anisotropic nonlinear material parameter characteristics, respectively [14]. For the cervical ligaments, different anatomical thicknesses were used according to the results of previous studies and were represented by orthogonal anisotropic nonlinear properties. The articular process cartilage was expressed by the linear elastic material with an elastic modulus of 10 MPa. See Table 1 for specific material properties.

### Boundary, contact, and loading conditions

In the model, the bottom surface of the T1 vertebra was completely constrained. An axial compression load of 73.6 N was used to simulate the head weight, which was applied to the tip of the C2 vertebral body. At the same time, the models of the three planes (flexion, extension, lateral bending and axial rotation) of the natural motion of the cervical vertebra were analyzed under the working condition that a torque of ± 2 N-m was applied on the top of the C2 vertebral body. Contacts between the cage screws and the endplate were modeled using surface-to-surface contact elements whose coefficient of friction was 0.5 [18]. There was a frictionless nonlinear contact relationship between the joints [19, 20], and all other contacts were set as bonded.

## Results

### Mesh sensitivity test

The FE model was tested for mesh sensitivity. Three mesh resolutions were generated consecutively (in the order of Mesh 1, Mesh 2, and Mesh 3) for this FE model. Mesh 3 had the smallest number of elements and nodes among the three mesh resolutions. Mesh 2 had approximately triple numbers of elements and nodes than the previous mesh resolution. The number of elements and nodes for each mesh resolution are shown in Additional file 1: Appendix table A, which shows that the difference of the results is less than 5%, and the mesh is convergent [21].

### Model validation

**Fig. 3:**
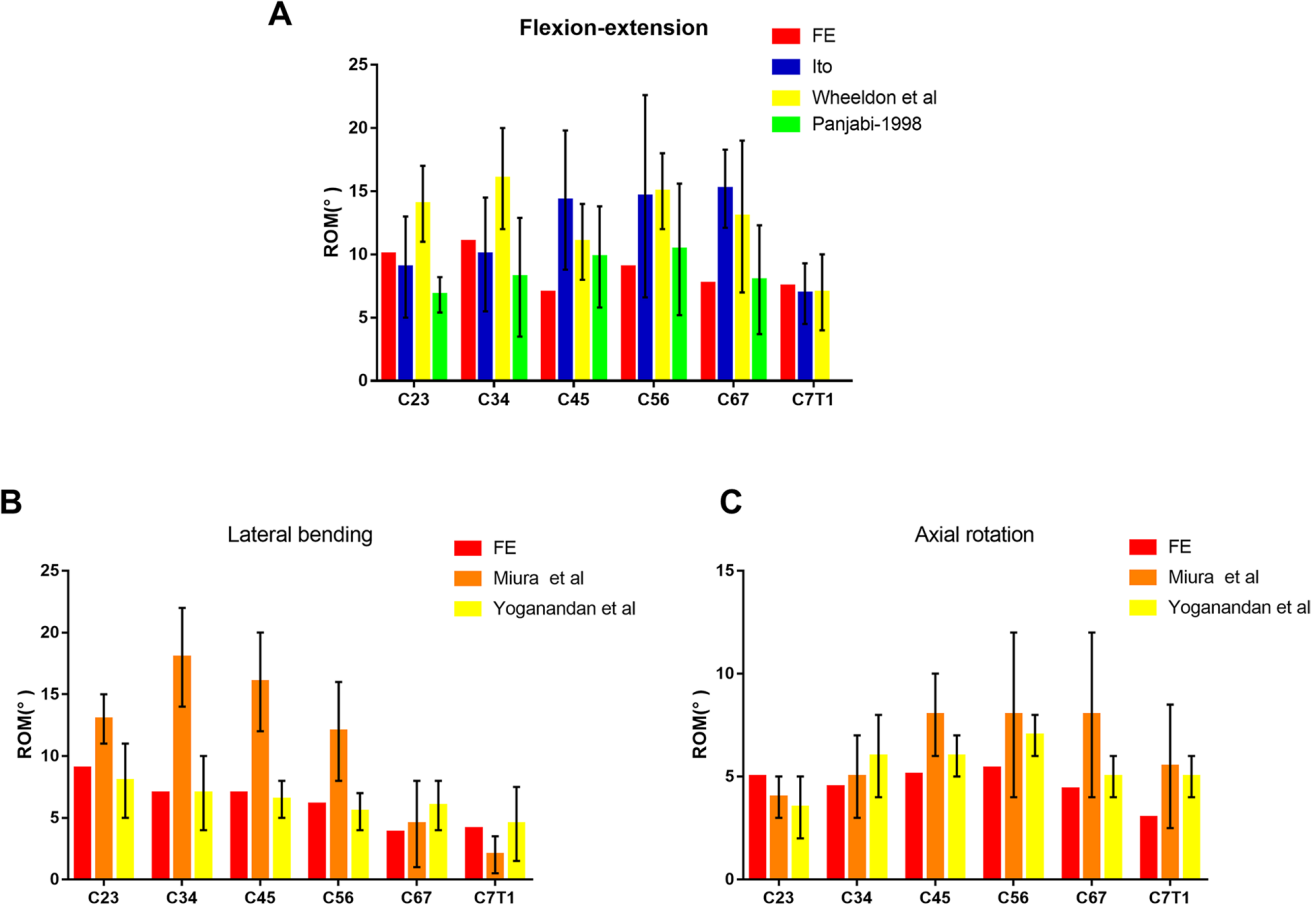
Comparison of the range of motion (ROM) of the intact FE models of C2**-**T1 with the previous biomechanical studies. **A** ROM in flexion–extension. **B** ROM in lateral bending. **C** ROM in axial rotation

The intact cervical C2–T1 model established in this study was compared with previously published in vitro experimental results to evaluate its effectiveness. The segmental ranges of motion (ROMs) of the FE model in flexion and extension, lateral bending and axial rotation were all within the range of the results observed in the previous experiments and the FE research [22–26]. The three-dimensional FE model established in this study was validated. See Fig. 3 for details.

### ROM

**Fig. 4:**
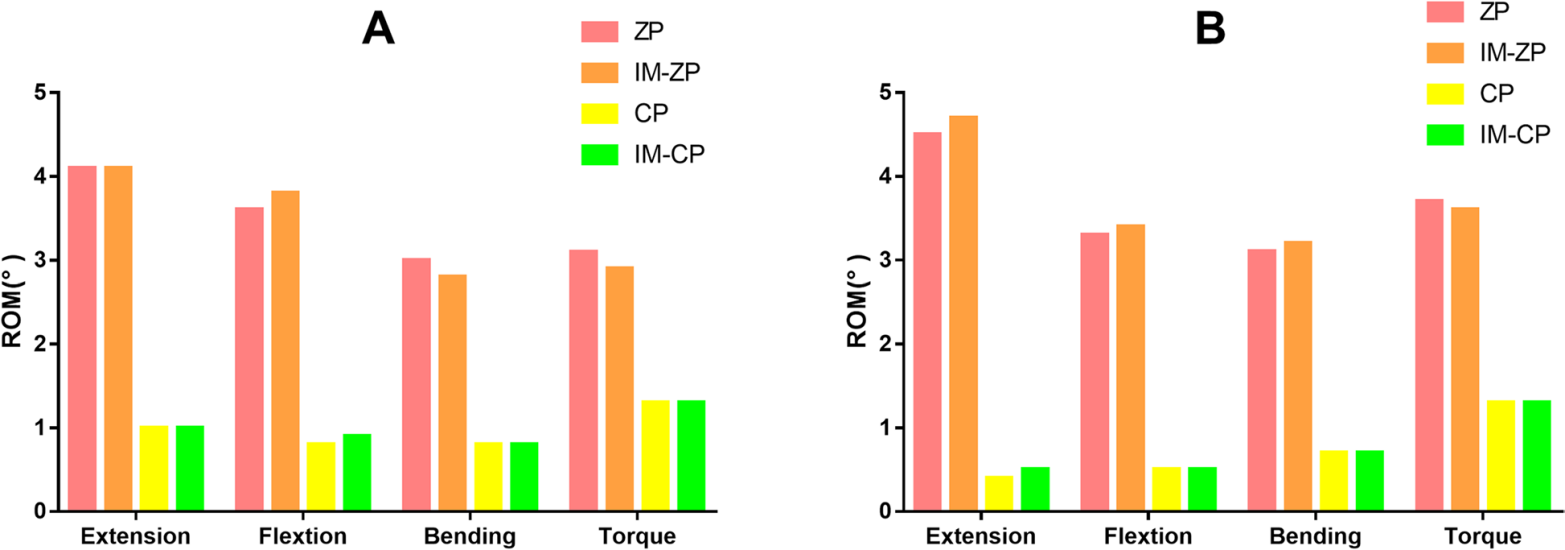
Comparison of the range of motion (ROM) of the four FE models (ZP, IM-ZP, CP, IM-CP).  **A** The comparison of ROM in C4/5.  **B** The comparison of ROM in C5/6

Figure 4 compares the ROMs of the operated segments (C4/5 and C5/6) of the four mechanical models. When the ZP model and the IM-ZP model are compared, there is no obvious difference in the segmental ROM comparison between them under the working conditions of flexion and extension, lateral bending and axial rotation. The ROMs of two segments (C4/5, C5/6) of the ZP model and the IM-ZP model are similar under the working conditions of flexion and extension, lateral bending and rotation. When the CP model and the IM-CP model are compared, there is no obvious difference between them in the segmental ROM comparison under the working conditions of flexion and extension, lateral bending and axial rotation. The ROMs of two segments (C4/5 and C5/6) of the CP model and the IM-CP model are similar under the working conditions of lateral bending and rotation, while the ROMs of the C4/5 segments of the CP model and IM-CP model under the working conditions of forward flexion and backward extension (CP: forward flexion: 0.8°, backward extension: 1.0°; IM-CP: forward flexion: 0.9°, backward extension: 1.0°) are significantly higher than those of the C5/6 segments (CP: forward flexion: 0.4°, backward extension: 0.5°; IM-CP: forward flexion: 0.5°, backward extension: 0.5°). When the ZP model is compared with the CP model under the working conditions of flexion and extension, lateral buckling and rotation, the ROMs of the ZP model are obviously greater than those of the CP model. This is also the case with the comparison between the IM-ZP model and the IM-CP model.

**Fig. 5:**
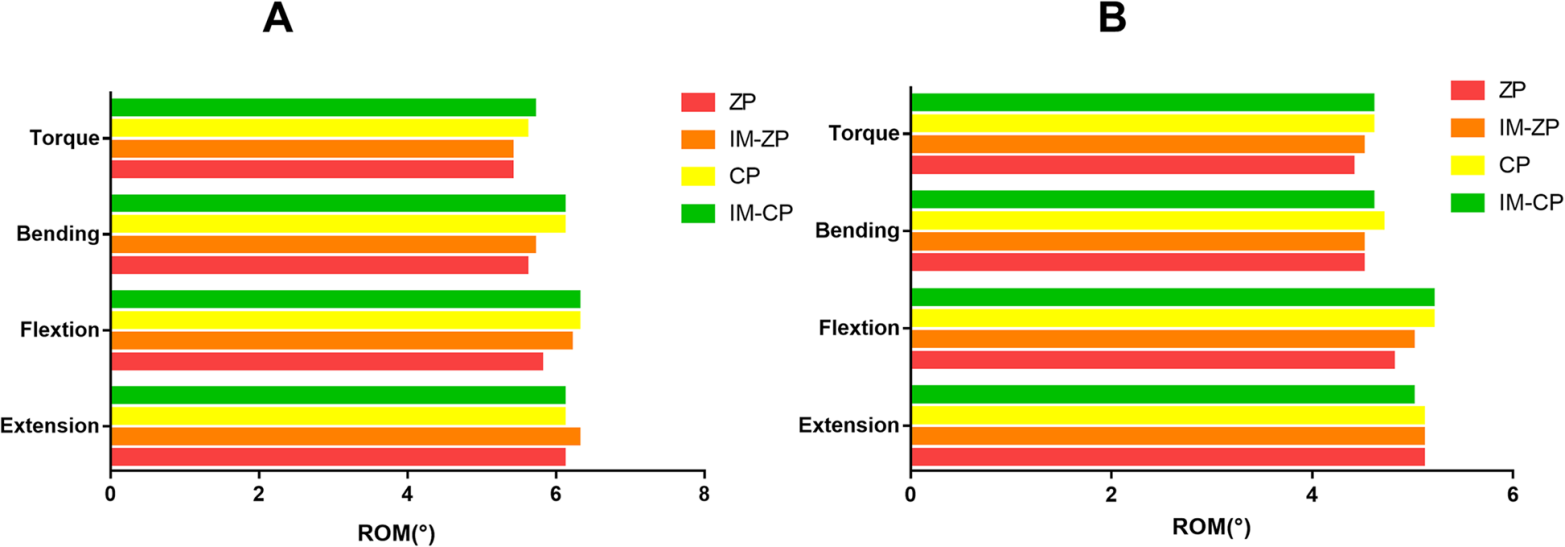
Comparison of the range of motion (ROM) of the adjacent segments in four FE models (ZP, IM-ZP, CP, IM-CP).  **A** The comparison of ROM in C3/4. **B** The comparison of ROM in C6/7

The results in Fig. 5 and Table 3 indicate that the four models have little difference in the ROMs between adjacent segments. When the ZP model and the CP model are compared, the ROMs of the adjacent segments of the ZP model are slightly lower than those of the CP model under the working conditions of forward flexion, lateral bending and axial rotation. This is also the case with the comparison between the IM-ZP model and the IM-CP model. The ROMs of the adjacent segments of the ZP model under the working condition of backward extension are slightly higher than those of the CP model, and the ROMs of the adjacent segments of the IM-ZP model are also slightly higher than those of the ZP model. When the ZP model and the IM-ZP model are compared, the ROMs of adjacent segments of the IM-ZP model under the working conditions of flexion and extension are slightly increased compared with those of the ZP model. The ROMs of the C3/4 and C6/7 segments in both models under the working conditions of flexion and extension are significantly higher than those in other directions. When the CP model and the IM-CP model are compared, the ROMs of adjacent segments in these two models are essentially the same.

**Table 3:** Comparison of adjacent segments range of motion (ROM) after two-level ACDF using cage plus plate (CP) or zero-profile device (ZP)

ROM (°)	ZP		IM-ZP		CP		IM-CP	
	C34	C67	C34	C67	C34	C67	C34	C67
Extension	6.1	5.1	6.3	5.1	6.1	5.1	6.1	5.0
Flextion	5.8	4.8	6.2	5	6.3	5.2	6.3	5.2
Bending	5.6	4.5	5.7	4.5	6.1	4.7	6.1	4.6
Torque	5.4	4.4	5.4	4.5	5.6	4.6	5.7	4.6

### Von Mises stress

#### Comparison of endplate stress (MPa)

**Fig. 6:**
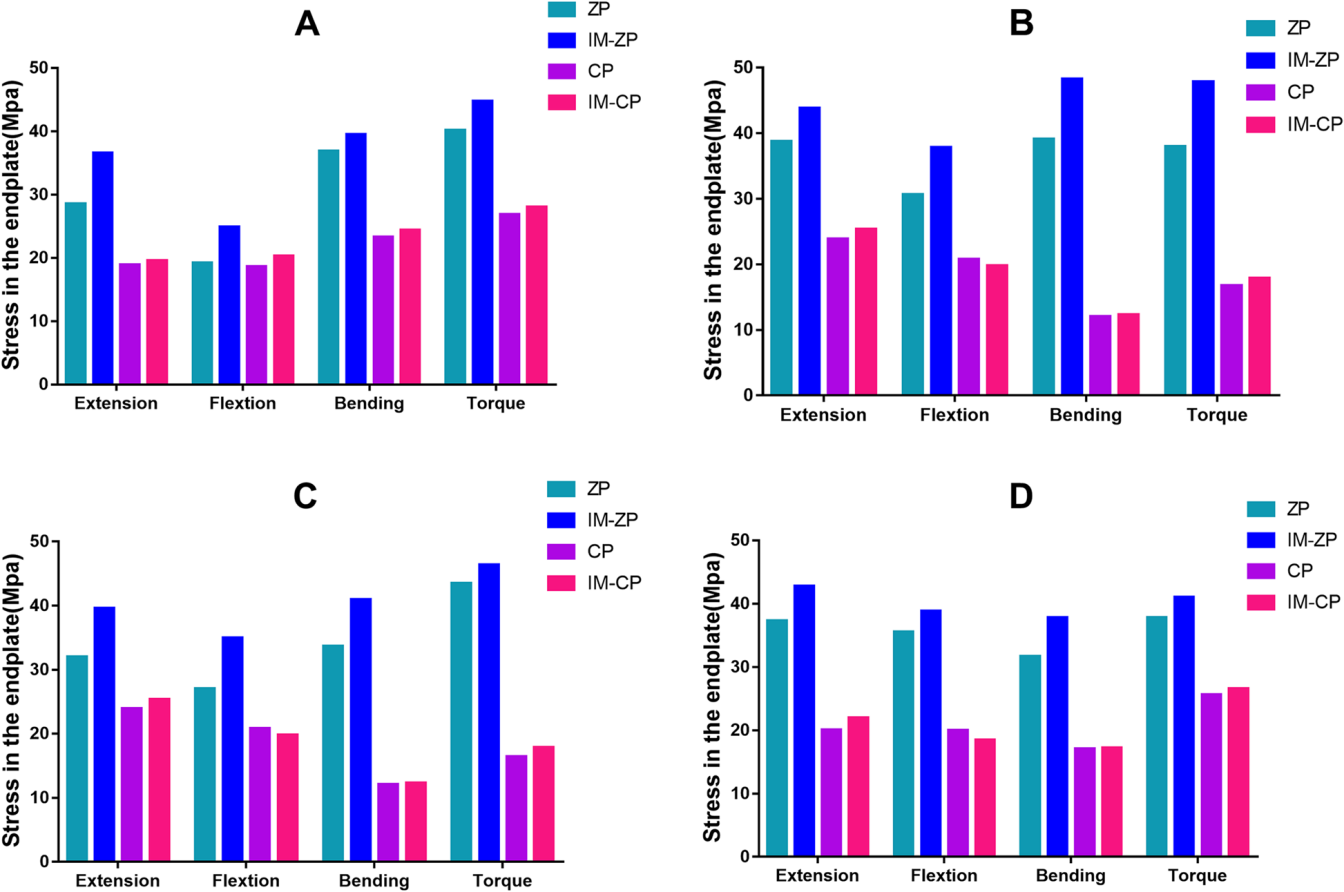
Comparison of the endplate stress of the C4/5 and C5/6 segments in four FE models (ZP, IM-ZP, CP, IM-CP). **A** The comparison of stress in lower endplate of C4. **B** The comparison of stress in upper endplate of C5. **C** The comparison of stress in lower endplate of C5. **D** The comparison of stress in upper endplate of C6

**Fig. 7:**
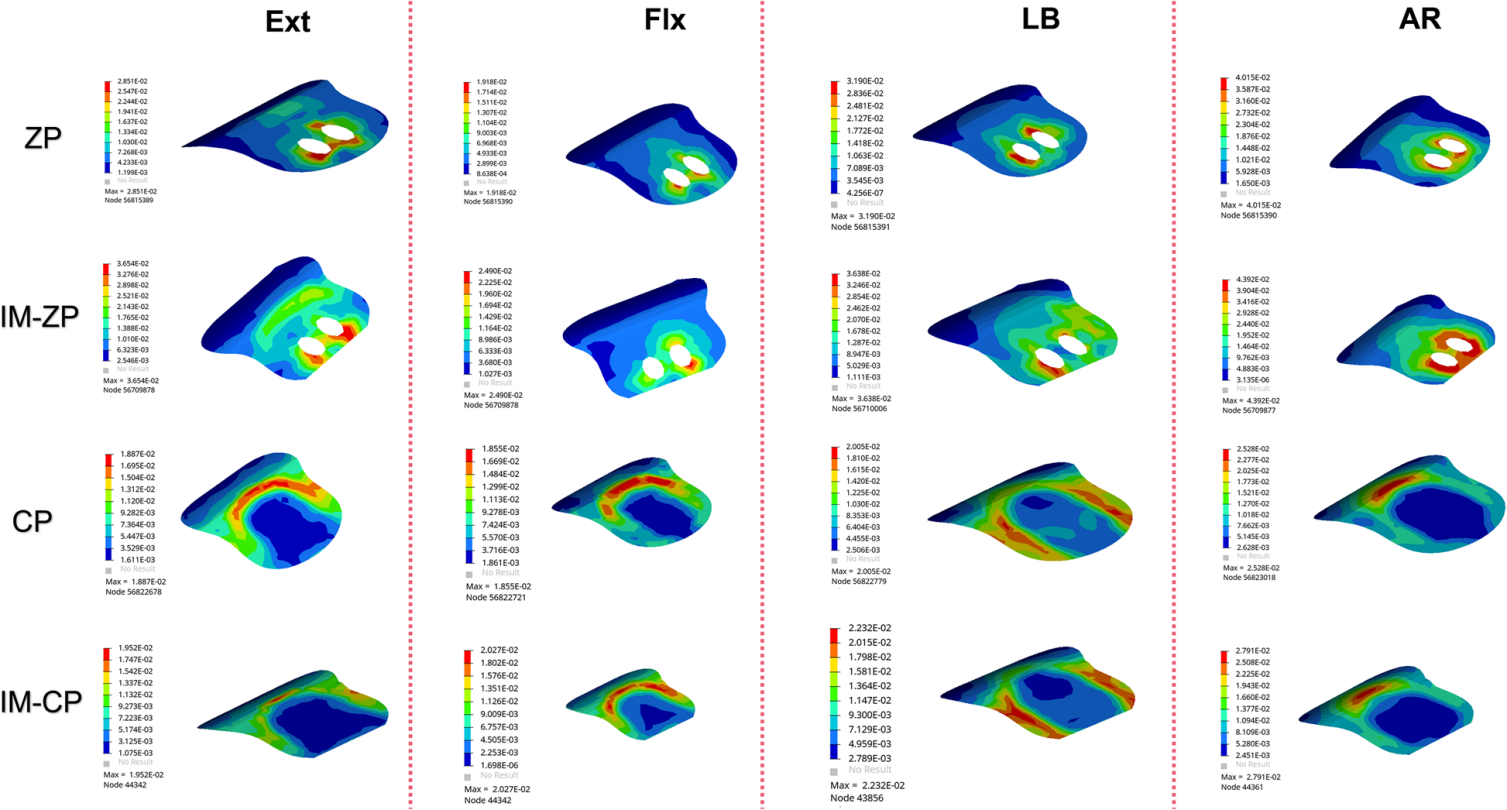
Stress distribution nephograms of the lower endplate of C4 in both groups. Ext: Extension; Flx: Flexion; LB: Lateral bending; AR: Axial rotation

**Fig. 8:**
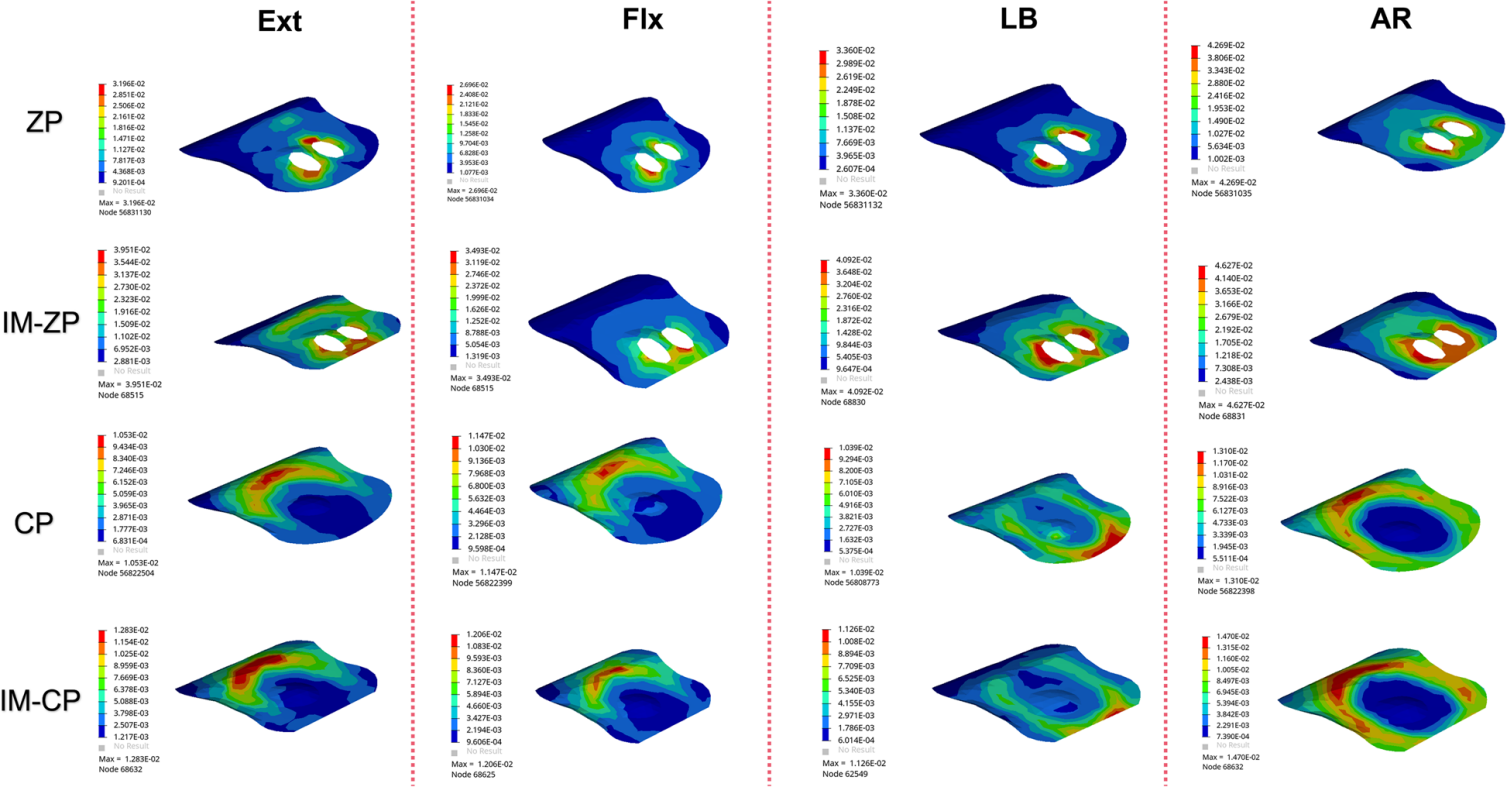
Stress distribution nephograms of the lower endplate of C5 in both groups

Figure 6 and Table 4 show the stress comparison of the upper and lower endplates of the surgical segments (C4/5 and C5/6) of the four mechanical models under the working conditions of forward flexion, backward extension, lateral bending and axial rotation. Under the working conditions of forward flexion and backward extension, the stress on the C5 lower endplate in the four mechanical models is considerably greater than the stress on the C4 lower endplate. When the ZP model and the CP model are compared, the endplate stresses of the ZP model are obviously higher than those of the CP model under the working conditions of forward flexion, backward extension, lateral bending and axial rotation. When the ZP model and the IM-ZP model are compared, the endplate stresses of the IM-ZP model under the working conditions of forward flexion, backward extension, lateral bending and axial rotation are greater than those of the ZP model, with the stress of the C4 lower endplate being 23.0%, 22.0%, 6.6% and 10.2% greater, respectively; the stress of the C5 upper endplate being 19.0%, 11.6%, 18.9% and 20.7% greater, respectively; the stress of the C5 lower endplate being 22.8%, 19.1%, 17.9% and 6.2% greater, respectively; and the stress of the C6 upper endplate being 8.4%, 12.7%, 16.2% and 7.8% greater, respectively. The stress distribution nephogram shows that the endplate stresses of both the ZP model and the IM-ZP model are concentrated at the first one-third of the endplates, and the stress at the junction with the screw is the highest (Figs. 6 and 7). When the CP model and the IM-CP mode are compared, the endplate stresses of the two models are very close to each other under the working conditions of forward flexion, backward extension, lateral bending and axial rotation, with the endplate stress of the IM-CP model being slightly greater than that of the CP model, but there is no clinical difference (Fig. 5). The stress distribution nephogram shows that the endplate stresses of the two models are evenly distributed on the endplates. Under the working conditions of flexion, extension and axial rotation, the highest stress occurs at the contact surface between the last one-third of the endplates and the fusion S, and under the working condition of lateral bending, the highest stress occurs at both lateral sides of the endplates (Figs. 7 and 8).

**Table 4:** Comparison of endplate pressure after two-level ACDF using CP or zero-profile device ZP

FE	Extension				Flexion				Lateral bending				Axial			
	ZP	IM-ZP	CP	IM-CP	ZP	IM-ZP	CP	IM-CP	ZP	IM-ZP	CP	IM-CP	ZP	IM-ZP	CP	IM-CP
A	28.51	36.54	18.87	19.52	19.18	24.9	18.55	20.27	36.85	39.45	23.27	24.33	40.15	44.71	26.83	27.99
B	38.73	43.79	23.85	25.30	30.61	37.8	20.76	19.74	39.10	48.23	12.03	12.31	37.93	47.82	16.72	17.84
C	31.96	39.51	23.85	25.30	26.96	34.93	20.76	19.74	33.60	40.92	12.03	12.31	43.44	46.30	16.42	17.81
D	37.32	42.77	20.04	21.93	35.53	38.78	19.94	18.4	31.64	37.75	17.08	17.19	37.84	41.04	25.59	26.55

*A* The inferior endplate of C4, *B* The superior endplate of C5, *C* The inferior endplate of C5, *D* The superior endplate of C6

#### Comparison of screw stress (MPa)

**Fig. 9:**
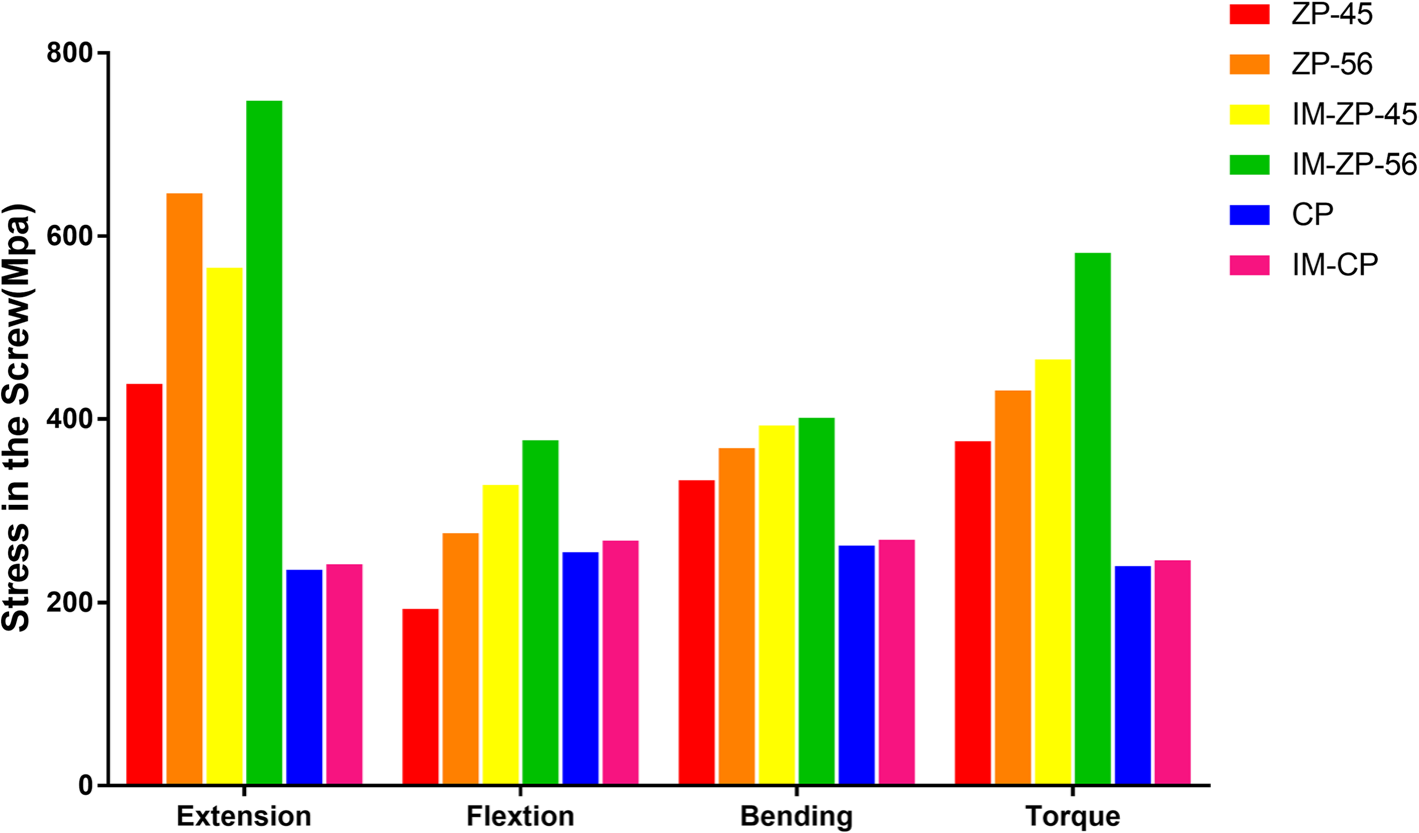
Comparison of the screw stress of the C4/5 and C5/6 segments in four FE models (ZP, IM-ZP, CP, IM-CP). ZP-45: The screw stress of C4/5 in ZP model; ZP-56: The screw stress of C5/6 in ZP model; IM-ZP-45: The screw stress of C4/5 in IM-ZP mode; IM-ZP-56: The screw stress of C5/6 in IM-ZP model

**Fig. 10:**
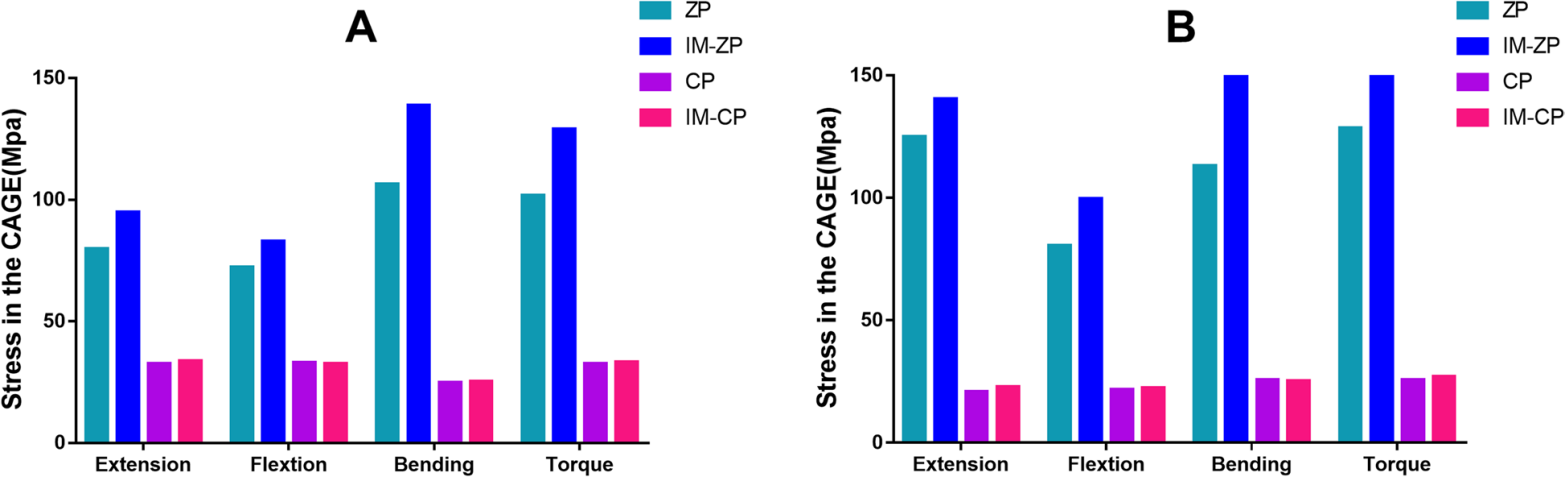
Stress distribution nephograms of the fixation device in both groups. Ext: Extension; Flx: Flexion; LB: Lateral bending; AR: Axial rotation

Figure 9 and Table 5 show the stress comparison of screws of the surgical segments (C4/5 and C5/6) in the four mechanical models under the working conditions of forward flexion, backward extension, lateral bending and axial rotation. When the ZP model and the CP model are compared, the fixation stresses in the ZP model are obviously greater than those in the CP model under the working conditions of backward extension, lateral bending and axial rotation. When the IM-ZP model and the IM-CP model are compared, under the six working conditions, the fixation stresses in the IM-ZP model are obviously greater than those in the IM-CP model. Moreover, the fixation stresses in either the ZP model or the IM-ZP model are mainly concentrated in the screws, with the stress at the junction between the screw and the cage being the largest, while the fixation stresses in the CP model and the IM-CP model are distributed evenly on the steel plates (Fig. 10). When the ZP model and the IM-ZP model are compared, the screw stresses in the IM-ZP model under the working conditions of flexion, extension, lateral bending and axial rotation are greater than those in the ZP model. The stresses of the C5/6 screws of the two models are greater than those of the C4/5 screws under all of the working conditions. When the screw stresses are compared under the working conditions of forward bending, lateral bending and axial rotation, the screw stresses in both models are the largest under the backward extension condition. In comparison with the ZP model, under the working conditions of forward flexion, backward extension, lateral bending and axial rotation, the stresses of the C4/5 screws in the IM-ZP model are 41.2%, 22.4%, 15.1% and 19.1% greater, respectively, and the stresses of the C5/6 screws in the IM-ZP model are 26.9%, 13.5%, 8.1% and 25.9% greater, respectively. When the CP model and the IM-CP model are compared, under the working conditions of forward flexion, backward extension, lateral bending and axial rotation, the stresses of the internal fixation device in the IM-CP model are slightly greater than those in the CP model, and there is no clinical difference (Fig. 8).

**Table 5:** Comparison of screw pressure after two-level ACDF using CP or zero-profile device ZP

Screw	ZP		IM-ZP		CP	IM-CP
	C45	C56	C45	C56	-	-
Extension	438.60	646.60	565.30	747.90	236.00	242.10
Flextion	193.20	276.00	328.70	377.60	255.30	267.80
Bending	333.70	369.10	393.20	401.60	262.40	268.70
Axial rotation	376.30	431.40	465.30	581.80	240.00	246.40

#### Comparison of cage stress (MPa)

**Fig. 11:**
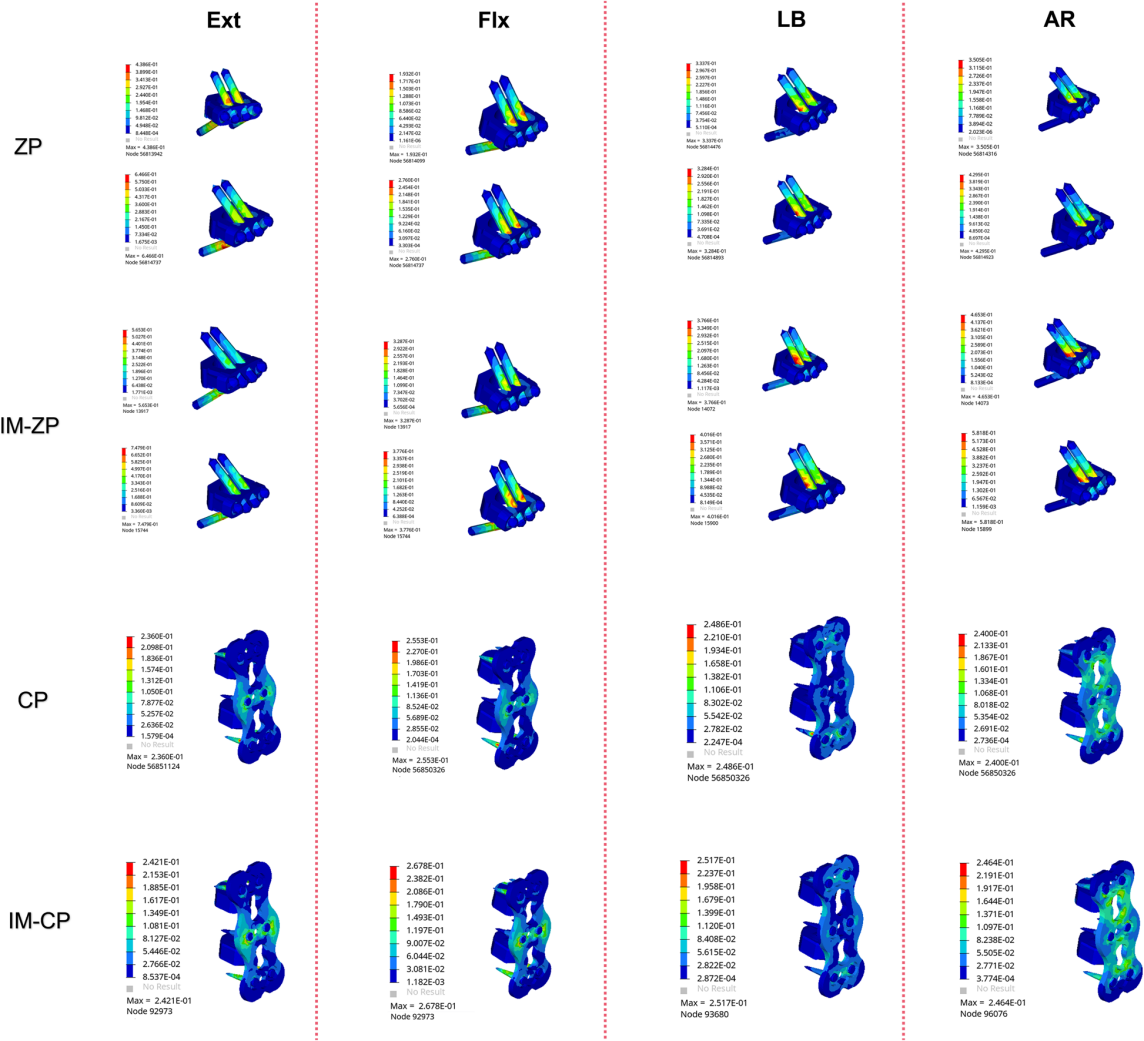
Comparison of the cage stress of the C4/5 and C5/6 segments in four three-dimensional finite element models (ZP, IM-ZP, CP, IM-CP). A: The comparison of cage stress in C4/5; B: The comparison of cage stress in C5/6

Figure 11 and Table 6 show the cage stress comparison of the surgical segments (C4/5 and C5/6) in the four mechanical models under the working conditions of forward flexion, backward extension, lateral bending and axial rotation. In the comparison with the CP model, the cage stresses in the ZP model are significantly higher than those in the CP model under the six working conditions, and this is also the case with the comparison between the IM-ZP model and the IM-CP model. The ROMs of the ZP model are significantly greater than those of the CP model under the working conditions of flexion, extension, lateral bending and rotation. When the ZP model and the IM-ZP model are compared, the cage stresses in the IM-ZP model are higher than those of the ZP model under the working conditions of forward flexion, backward extension, lateral bending and axial rotation. The cage stresses of C5/6 in the two models are higher than those of C4/5 under the six working conditions. In the comparison of the two models under the working conditions of forward flexion, backward extension, lateral bending and axial rotation, the fusion case stresses in the two models are the highest under the lateral bending condition. In comparison with the ZP model, under the working conditions of forward flexion, backward extension, lateral bending and axial rotation, the stresses of the C4/5 cage in the IM-ZP model are 12.8%, 15.9%, 23.3% and 21.1% greater, respectively, and the stresses of the C5/6 cage are 19.1%, 11.0%, 39.0% and 18.7% greater, respectively. When the CP model and the IM-CP model are compared, the cage stresses in the IM-CP model are similar to those in the CP model under the working conditions of forward flexion, backward extension, lateral bending, and axial rotation (Fig. 11).

**Table 6:** Comparison of cage pressure after two-level ACDF using CP or zero-profile device ZP

Cage	ZP		IM-ZP		CP		IM-CP	
	C45	C56	C45	C56	C45	C56	C45	C56
Extension	79.80	124.90	94.85	140.40	32.60	20.81	33.54	22.72
Flextion	72.21	80.48	82.79	99.51	32.92	21.64	32.56	22.33
Bending	106.40	113.00	138.80	185.10	24.84	25.67	25.23	25.09
Axial rotation	101.80	128.40	129.00	157.90	32.40	25.62	33.27	26.88

#### Comparison of vertebral stress (MPa)

**Fig. 12:**
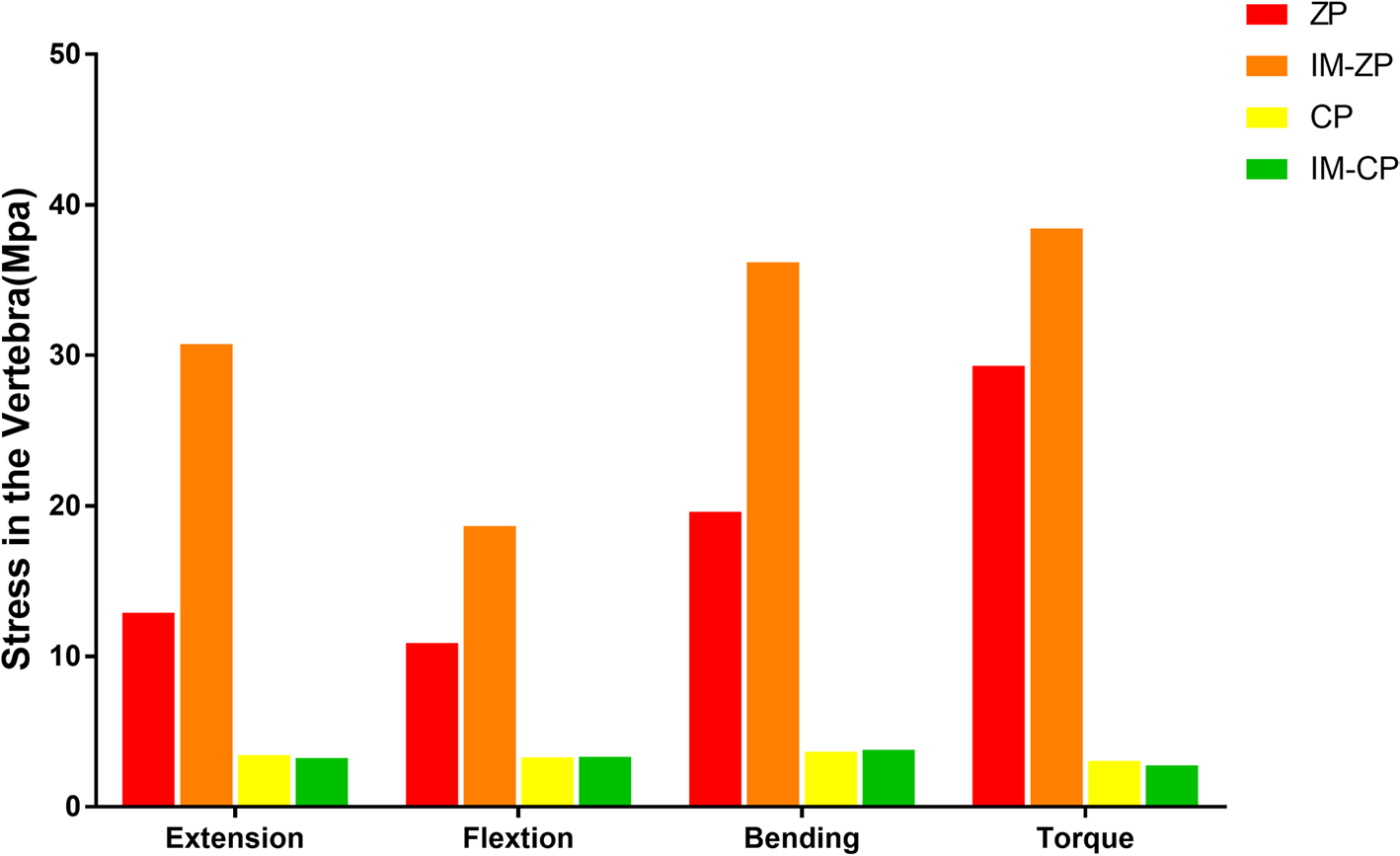
Comparison of the stress in C5 vertebrae in four FE models (ZP, IM-ZP, CP, IM-CP)

**Fig. 13:**
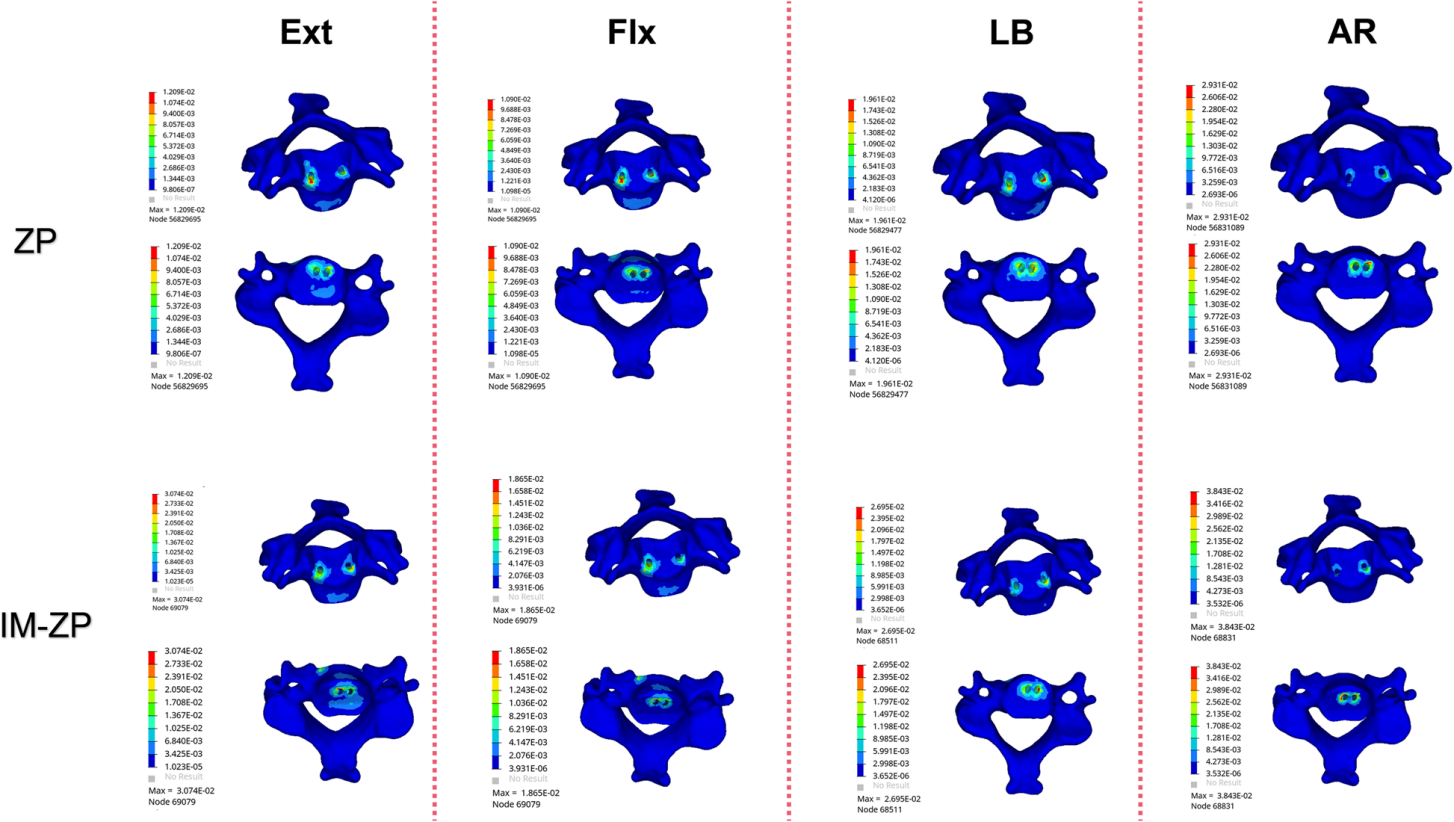
Stress distribution nephograms of the C5 vertebrae in ZP and IM-ZP models. Ext: Extension; Flx: Flexion; LB: Lateral bending; AR: Axial rotation

**Fig. 14:**
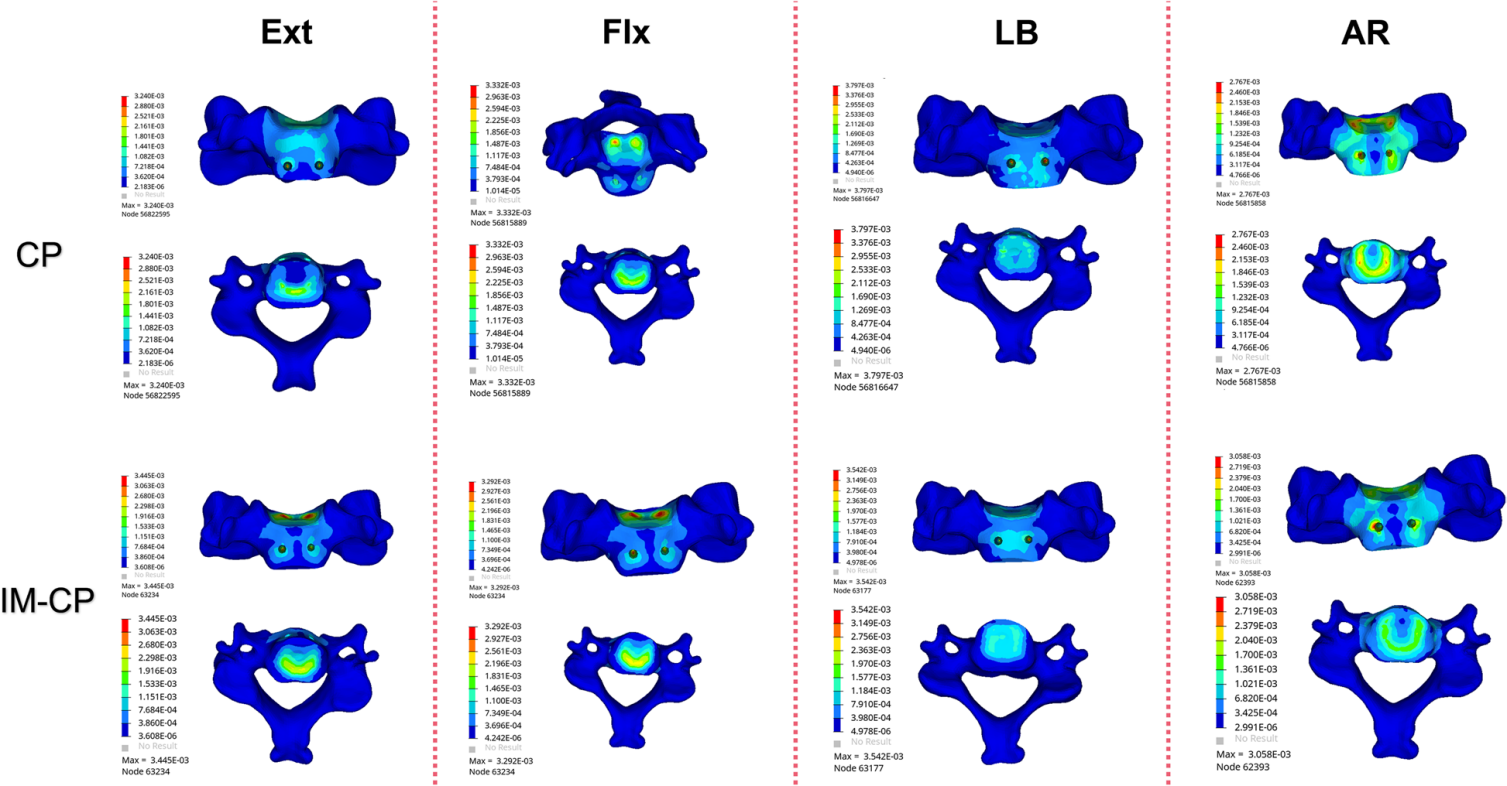
Stress distribution nephograms of the C5 vertebrae in CP and IM-CP models. Ext: Extension; Flx: Flexion; LB: Lateral bending; AR: Axial rotation

Figure 12 and Table 7 shows the stress comparison of the cancellous bones in the middle vertebral body (C5) of the surgical segments in the four mechanical models under the working conditions of forward flexion, backward extension, lateral bending and axial rotation. When the ZP model and the IM-ZP model are compared, the stresses of the cancellous bone in the C5 vertebral body of the IM-ZP model under the working conditions of forward flexion, backward extension, lateral bending and axial rotation are greater than those of the ZP model. In the comparison under the working conditions of forward flexion, backward extension and lateral bending, the stresses of the C5 vertebral bodies of the two models are the largest under the working condition of axial rotation. In comparison with the ZP model, in the IM-ZP model, the stresses of the cancellous bone in the C5 vertebral body are 41.6%, 58.0%, 45.8% and 23.7% greater under the working conditions of forward flexion, backward extension, lateral bending and axial rotation, respectively. The stress distribution nephogram shows that the stresses of the ZP model and the IM-ZP model are concentrated in the first one-third of the vertebral body and are the largest at the junction between the screw and the vertebral body (Fig. 13). When the CP model and the IM-CP model are compared, under the working conditions of forward flexion, backward extension, lateral bending and axial rotation, the stresses of the cancellous bone in the C5 vertebral body of the IM-CP model are similar to those of the CP model (Fig. 12 and Table 7). The stresses of the cancellous bone in the C5 vertebral body of the CP model and the IM-CP model are mainly distributed at the posterior side and both lateral sides of the vertebral body (Fig. 14). In the comparison among the four mechanical models, the stresses of the C5 vertebral bodies of the zero-profile models are obviously higher than those of the steel-plate models under the six working conditions.

**Table 7:** Comparison of pressure in C5 after two-level ACDF using CP or zero-profile device ZP

	ZP	IM-ZP	CP	IM-CP
Extension	12.90	30.74	3.44	3.24
Flextion	10.90	18.65	3.29	3.33
Bending	19.61	36.18	3.66	3.79
Axial rotation	29.31	38.43	3.05	2.76

#### Comparison of intradiscal pressure (MPa)

**Fig. 15:**
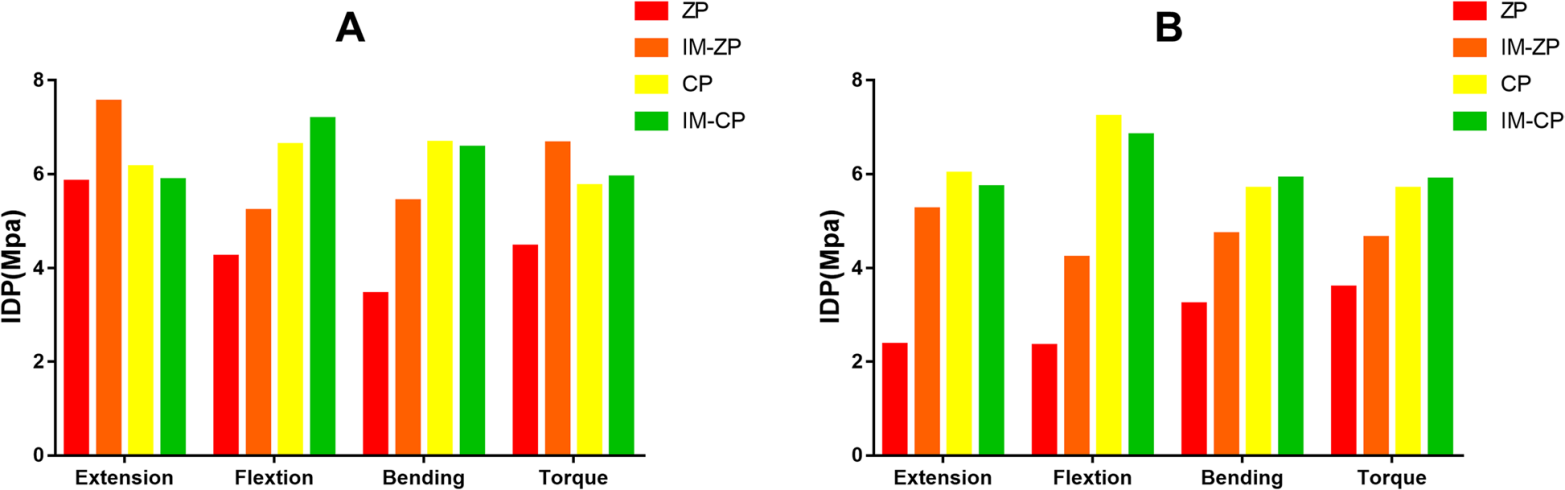
Comparison of the intradiscal pressure of the C3/4 and C6/7 segments in four FE models (ZP, IM-ZP, CP, IM-CP). A: The comparison of IDP in C3/4; B: The comparison of IDP in C6/7

When the ZP model and the CP model are compared, the intradiscal pressure (IDP) values of the adjacent segments in the ZP model are smaller than those in the CP model under the six working conditions (Fig. 15 and Table 8). When the IM-ZP model and the IM-CP model are compared, under the working conditions of forward flexion and lateral bending, the IDPs of the adjacent segments in the IM-ZP model are all smaller than those in the IM-CP model. Under the working conditions of backward extension and rotation, the IDPs of the C3/4 segment in the IM-ZP model are higher than those in the IM-CP model, while the IDPs of the C6/7 segment in the IM-ZP model are smaller than those in the IM-CP model. In the comparison between the ZP model and the IM-ZP model, under forward flexion, backward extension, lateral bending and axial rotation, the IDPs of the adjacent segments (C3/4, 6/7) in the IM-ZP model are all greater than those in the ZP model. In the comparison between the ZP model and the IM-ZP model, under the working conditions of forward flexion, backward extension, lateral bending and axial rotation, respectively the IDPs of the C3/4 segment in the IM-ZP model are 18.7%, 22.6%, 36.5% and 33.1% greater than those in the ZP model, and the IDPs of the C6/7 segment are 44.5%, 55.1%, 31.6%, and 22.7% greater than those in the ZP model (Fig. 15). There is no significant difference in the IDPs of the adjacent segments (C3/4, 6/7) between the CP model and the IM-CP model. In the comparison among the four mechanical models, the IDPs of the adjacent segments (C3/4, 6/7) in the steel-plate fixation models are higher than those in the zero-profile fixation models except for the case of the C3/4 segment in the IM-ZP model under the working condition of backward extension.

**Table 8:** Comparison of cervical intradiscal pressure after two-level ACDF using CP or ZP

Cage	ZP		IM-ZP		CP		IM-CP	
	C34	C67	C34	C67	C34	C67	C34	C67
Extension	5.83	2.36	7.54	5.25	6.14	6.01	5.86	5.72
Flextion	4.24	2.34	5.21	4.21	6.62	7.21	7.16	6.82
Bending	3.44	3.22	5.42	4.71	6.67	5.68	6.55	5.91
Axial rotation	4.45	3.59	6.65	4.64	5.74	5.69	5.93	5.88

## Discussion

There has been no clear conclusion about which fixation method is better, ZP or CP, in two-level ACDF operations. From the perspective of mechanical conduction, in the ZP fixation model, the ZP screw and cage are integrated; thus, the force transmission is greater through the vertebra-screw and cage interface during cervical spine movement. In our ZP model, it can be seen from the stress distribution nephogram of the internal fixation device (Fig. 11) that the stress in the ZP model is mainly borne by the cage and the screw, and the stress on the screw is markedly greater in this model than in the CP model. The stress is concentrated at the contact interface among the cage, screw and vertebral body. The ZP is used in two-level ACDF, and the middle vertebral body is at the joint of the upper and lower ZPs. The stress is transmitted from the upper ZP to the middle vertebral body via the endplate and then to the lower ZP via the lower endplate. The CP fixation adopts the screw-fixed cage and steel plate. The anterior plate screw can support the fusion segment during cervical flexion and play the role of tension band during cervical backward extension. When the cervical spine rotates, the locking device between the steel plate and the screw can provide anti-rotation torsion. Therefore, we can see the stress of the middle vertebral body in the ZP model under various working conditions, and the stress of each endplate is obviously greater than that in the CP model (Table 3). From the stress distribution nephogram of the internal fixation device in the CP model (Fig. 11), we can see that the stress is uniformly distributed on the steel plate-screw, while the cage bears little stress. Less force is conducted through the cage-vertebra interface. The ZP application in two-level ACDF objectively puts forward higher requirements for the strength of the endplate. In the case of insufficient endplate support strength, the ZP model is more likely to have complications such as endplate fracture and cage sinking. Although cage sinking may be asymptomatic, it may also lead to problems such as intervertebral foramen stenosis and nerve root compression.

Previous studies [27] indicate that the micromotion of the fusion levels can aggravate the instability of such segments. The instability of fusion segments may lead to the prolongation of the intervertebral fusion time and the sinking of the cage [28]. The stability of the fixed segments is an important factor affecting the prognosis. In this study, in the comparison between the CP model and the ZP model, the ROMs of the fusion segments of the ZP model are obviously greater than those of the CP model under the working conditions of flexion, extension, lateral bending and rotation. Under various working conditions, the ROMs of the fusion segments of the ZP model are approximately 3 degrees (up to 4 degrees in backward extension). In the CP model, the ROMs of the fusion segments are only 1 degree under various working conditions. Although FE modeling simulates the real situation as closely as possible, some elements still differ between the model and the real situation. Therefore, the ROM value of the fixed segment is used only as a reference. However, we can also see a trend from this. That is, although the cage and screw in the ZP model bear a greater stress, they have insufficient restrictions on the degree of segmental motion compared with the anterior cervical plate. The CP model can better restrict the activity of fusion segments and therefore make the fixation more stable. The residual ROM of fusion segments can easily cause micromotion of the fusion segments when the cervical vertebrae are moving in any direction. Excessive micromotion will make it difficult to stably couple the endplate-cage interface. Because of instability, the cage may have slight displacement, which further interferes with intervertebral fusion. Intervertebral fusion is thus delayed, and the long-term dynamic stimulation of the endplate by the cage may lead to endplate fracture and cage sinking. On the other hand, unstable micromotion will also cause the static system, including the cervical vertebrae, ligaments and intervertebral disc, to be unstable, and then the biomechanical imbalance of cervical vertebrae can also cause the cage to sink, increasing the risk of the collapse of the intervertebral space and vertebral body.

In ACDF, it is important to protect the integrity of the bone endplate as much as possible during the operation. Damage to the endplate will affect its supporting strength to the cage. Therefore, care should be taken to avoid excessive decortication of the cartilage endplate when scraping it during surgery. However, at the same time, due to the degeneration and proliferation of the vertebral body, osteophytes are formed. The anterior and inferior margins of the cervical vertebra often develop beak-like structures and cover the intervertebral disc below. Removal of such osteophytes is a routine procedure in ACDF surgery. In practice, there are morphological differences among the endplates. The upper and lower endplates of the intervertebral space are not perfectly parallel, and the upper endplate of the intervertebral space is often concave into the vertebral body, while the lower endplate is relatively flat. The intervertebral space is widest in the central part of the vertebral body but is narrow at the anterior and posterior margins. To obtain an adequate field of view and to insert a cage with almost parallel upper and lower surfaces, it is necessary to remove part of the bone at the anterior and inferior margins of the upper vertebral body. In some cases, too much bone has to be removed at this site, resulting in the separation of the anterior vertebral cortex and the endplate cortex and the exposure of the cancellous bone at this site, which may lead to defects in the anterior endplate bone. It can be seen from the comparison between the ZP model and the CP model that more stress in the ZP group is conducted via the endplate, and it is more important for the application of ZP in ACDF that the endplate has sufficient strength. Therefore, we constructed the IM-ZP model and the IM-CP model by simulating the anterior margin defect of the upper endplate of the intervertebral space caused during the operation.

According to the FE analysis in this study, in the comparison between the IM-ZP model and the ZP model, the stress of the endplate (Fig. 5), the stress of the internal fixation device (Figs. 8 and 9) and the stress of the cancellous bone in the middle vertebral body (Fig. 10) in the IM-ZP model were significantly greater under various working conditions, while the ROMs of the fusion segments of the two models were basically the same. The comparison of the results of the ZP and IM-ZP models showed that although the anterior endplate defect in the intervertebral space did not result in any clinically significant increase in the ROM of the fusion segment, the stresses of the endplate and internal fixation device of the fusion segment thus increased significantly. However, no similar situation was observed between the CP and IM-CP models. There was little difference between the two models in the ROMs of the fusion segments and the stresses of the endplates, the internal fixation devices and the cancellous bones in the middle vertebral bodies, which had no clinical significance. The steel plate was connected to the adjacent vertebral body with straight locking screws, providing a strong pre-tension band and strong rigid fixation and providing superior support and fixation. The ZP system merely provided fixation between the segments [29]. The stress distribution nephogram (Fig. 11) showed that in the CP and IM-CP models, the steel plate-screw bore the main load, and the stress distribution was uniform; the stresses in the ZP and IM-ZP models were concentrated at the junction among the screw, cage and endplate (Figs. 6 and 7). Previous studies [30] have shown that the contact junction between the endplate and the cage is also the concentration point of high stress (Figs. 6 and 7). The area with the highest thickness and strength of the cervical endplate is located in the posterior area of the upper endplate and the anterior area of the lower endplate, and the thinnest area is located in the central area [11, 31]. At the same time, the contact area is also an important determinant of the stress level [32]. Scholz et al. [33] found in an in vitro study that the ZP system is less stable than the CP plate system. Therefore, when the anterior margin of the endplate in the intervertebral space is defective, the contact between the internal fixation device of the ZP system and the endplate is reduced, resulting in greater changes in the mechanical parameters than those in the CP fixation system.

A previous study [33] showed that the vertebral body endplate can disperse pressure to the cancellous bone to enhance vertebral body stiffness. When the endplate is removed, the maximum compressive strength of the vertebral body will decrease by 33%. As shown in Fig. 10, with the change in endplate stress after endplate injury, the stress of the cancellous bone in the vertebral body also increases correspondingly. At the same time, as seen from the nephograms (Figs. 12 and 13), the stresses in the CP group models are mainly distributed on the posterior side and both lateral sides of the vertebral body, while those in the ZP group models are concentrated on the anterior side of the vertebral body, and the stress distribution under the C5 vertebral body is similar to that of the C5 lower endplate. Compared with the case of the CP group models, the stresses of the ZP group models on the cancellous bone of the C5 vertebral body are greater, and the stresses on the vertebral body of the ZP group models are concentrated on the anterior side. The anterior column of the vertebral body is the basic support structure of the spine, providing static stabilization and bearing 70% of the load. If the stress of the anterior side of the vertebral body increases and the endplate loses the support of the bone in the anterior wall of the vertebral body due to the defect, it will become more dependent on the support of the cancellous bone in the vertebral body. If the support of the cancellous bone is insufficient, long-term stress may lead to microfracture of the cancellous bone and eventually cause the endplate to collapse into the vertebral body. Lowe et al. [34] found in their in vitro experiments that subsidence of the implant is related to the biomechanical properties of the local area of the vertebral endplate and noted that the implant is the most resistant to subsidence when it is located on the posterior-lateral sides of the endplate, while the central area is the most prone to subsidence. Therefore, when the ZP is used in two-level ACDF, damage to the anterior edge of the bony endplate will lead to greater stresses on the endplate and cancellous bone in the vertebral body.

Adjacent segment degeneration (ASD) after fusion surgery is a major clinical problem after ACDF [35]. However, the cause of ASD is still controversial at present. Some experts believe that ASD is caused by the increased compensatory pressure of adjacent intervertebral discs during vertebral fusion surgery [36–38]. Adjacent segment intervertebral disc degeneration is a significant risk factor for ASD [39, 40]. A normal intervertebral disc can relieve shock and decompose stress, thus maintaining spinal stability. Abnormal intervertebral disc stress can lead to degeneration [41, 42]. In a previous FE study, Hua et al. [43] found that after two-level ACDF, the intervertebral disc pressures of the C3/4 and C6/7 segments were higher than those of the normal model, regardless of the use of a ZP method or a steel plate, but there was no obvious pressure difference between the two groups of models. A similar conclusion was also reached by Wu et al. [44] in an FE study. At the same time, it was also put forward that CP plate fixation may reduce the ROM of the surgical segment and increase the compensatory activity and IDP of adjacent segments, which can accelerate the degeneration process of the adjacent cervical spine of the fusion segment in coordination with natural aging and is more likely to develop ASD. In this study, the ROMs of the surgical segment of the CP group models were lower than those of the ZP group models (Fig. 4). With respect to the ROMs of adjacent segments, there was little difference among the four models, and there was no obvious clinical difference. In most cases, the IDPs of adjacent segments in the ZP application are significantly lower than those in the CP application, which is similar to the results of previous literature [45]. Only in the case of endplate damage is the IDP of the C3/4 segment higher under the working conditions of backward extension and rotation (Figs. 14 and 15). It is worth noting that in the ZP group models, although there was no difference in the adjacent segment ROM between the IM-ZP model and the ZP model, the adjacent segment IDP was significantly increased. This may be related to the increased stress on the adjacent vertebral body after endplate injury [46].

The limitations of the present study should be taken into account. The IVD is considered as a highly hydrated poroelastic tissue. However, the focus of this study is the biomechanical response of endplate defects on the intermediate vertebral bone in consecutive two-level ACDF, which is not predicting the post-yield behavior of the IVD. Many FE models have assumed that the components of the IVD are linear to improve the calculation efficiency [47–53]. The tendency of predicted results with various fixation options would not be substantially changed depending on the individual geometric model and simplified material properties. Further clinical studies evaluating the findings from this study are also expected in the future.

## Conclusions

Compared with CP fixation, ZP fixation in two-level ACDF can cause increased stress on the cancellous bone of the middle vertebral body. The application of ZP fixation in two-level ACDF relies more on the endplate to conduct stress, especially the anterior one-third of the endplate (Figs. 6 and 7). ZP fixation has higher requirements for end plate strength than CP fixation. Therefore, ACDF using ZP fixation requires more protection for the bony endplate, especially the anterior one-third. Because of the variations in endplate morphology due to cervical hyperplasia and degeneration, it is sometimes necessary in clinical practice to remove the anterior edge of the upper endplate of the fusion segment, resulting in a defect on the anterior edge of the bony endplate. This change compromises the support that the anterior wall of the vertebral body provides to the anterior one-third of the endplate, which requires more stress to be placed on the cancellous bone under the endplate. At the same time, the reduction in endplate area also increases the stress on the endplate. The upper and lower endplates of the middle vertebral body bear more stress than the lower endplate of the upper vertebral body, and there is no intervertebral disc to cushion and protect the joint. If any anterior edge defect is present at the same time, there is an increased risk that the cancellous bone under the endplate will be absorbed and remodeled due to insufficient support of the cancellous bone in the vertebral body, ultimately leading to the collapse of the vertebral body. Therefore, the application of CP devices is recommended during multilevel anterior cervical surgery for patients with endplate injuries.

## Supplementary Information

**Additinal file 1:** **Appendix table A**. Mesh sensitivity test.

## Data Availability

The data presented in this study are available on request from the corresponding author.
