# Supplementary material for: Biomechanical effect of endplate defects on the intermediate vertebral bone in consecutive two-level anterior cervical discectomy and fusion: a finite element analysis
Source: BMC Musculoskelet Disord. 2023 May 22;24:407. doi: 10.1186/s12891-023-06453-3 (PMC10201745; doi:10.1186/s12891-023-06453-3)
Supplement: Supplementary file 1 — Additinal file 1: Appendix table A. Mesh sensitivity test. [file 12891_2023_6453_MOESM1_ESM.docx]

**Appendix table A**

Mesh sensitivity test

| Mesh method | Mesh 1 | Mesh 2 | Mesh 3 |
| --- | --- | --- | --- |
| Size（mm） | 0.5 | 1 | 1.5 |
| Element number | 1826765 | 625444 | 385862 |
| Node number | 381709 | 114148 | 61910 |
| Cortical bone max stress (MPa) | 24.51 | 24.79 | 25.03 |
| Rate (change compared with mesh 1) |  | 1.14% | 2.12% |
| Cancellous bone max stress (MPa) | 2.44 | 2.53 | 2.39 |
| Rate (change compared with mesh 1) |  | 3.68% | 2.04% |
| Endplate max stress (MPa) | 18.01 | 17.49 | 17.73 |
| Rate (change compared with mesh 1) |  | 2.88% | 1.55% |
| Nucleus max stress (MPa) | 0.0185 | 0.0192 | 0.0177 |
| Rate (change compared with mesh 1) |  | 3.78% | 4.32% |
| Fiber max stress (MPa) | 0.0547 | 0.0526 | 0.0518 |
| Rate (change compared with mesh 1) |  | 3.83% | 5.31% |
| Facet max stress (MPa) | 0.0963 | 0.0917 | 0.0908 |
| Rate (change compared with mesh 1) |  | 4.7% | 5.71% |
